# Samsoeum water extract attenuates allergic airway inflammation via modulation of Th1/Th2 cytokines and decrease of iNOS expression in asthmatic mice

**DOI:** 10.1186/s12906-015-0561-3

**Published:** 2015-03-10

**Authors:** Woo-Young Jeon, In-Sik Shin, Hyeun-Kyoo Shin, Mee-Young Lee

**Affiliations:** Herbal Medicine Formulation Research Group, Korea Institute of Oriental Medicine, 483 Expo-ro, Yusung-gu, Daejeon, 305-811 Republic of Korea; Natural Medicine Research Center, Korea Research Institute of Bioscience and Biotechnology, 30 Yeongudanji-ro, O-chang uep, Cheongwon-gun, Cheongju-si, Chungbuk 363-883 Republic of Korea; Pharmacology, College of Veterinary Medicine, Chonnam National University, Gwangju, 500-757 Republic of Korea

**Keywords:** Samsoeum, Asthma, Inflammatory cells, Th1/Th2 cytokines, iNOS

## Abstract

**Background:**

Samsoeum has long been used in Korea and other Asian countries as a traditional medicine to treat various diseases. In the present study, we investigated the antiasthma effect of the herbal medicine Samsoeum water extract (SSEW) using an in vivo ovalbumin (OVA)-induced asthmatic model.

**Methods:**

Female BALB/c mice were sensitized by an intraperitoneal injection of OVA and subsequently challenged with nebulized OVA. We investigated the number of inflammatory cells, the production of Th1/Th2 cytokines and chemokine in bronchoalveolar lavage fluid (BALF), histological changes in lung tissue, the infiltration of inflammatory cells and hyperplasia of goblet cells in lung tissue, the levels of immunoglobulin E (IgE) in BALF and plasma, and the expression of inducible nitric oxide synthase (iNOS) in lung tissue.

**Results:**

Our findings indicated that SSEW decreased the accumulation of inflammatory cells (particularly, eosinophil and neutrophil) and regulated the balance in the production of Th1/Th2 cytokines and chemokine in BALF. Moreover, SSEW suppressed the level of IgE in BALF and plasma, and inhibited the infiltration of inflammatory cells, hyperplasia of goblet cells, and the expression of iNOS in lung tissue.

**Conclusions:**

Collectively, these results suggest that, because of its anti-inflammatory and antiasthma properties, SSEW may be useful in reducing airway inflammation in the treatment of allergic asthma.

## Background

Allergic asthma is a chronic inflammatory disease of the airway that is characterized by airway inflammation, intense eosinophilia, lymphocyte infiltration, and mucus overproduction [1]. Allergen-specific CD4^+^ T cells play a pivotal role in the development of asthma [2]. Th1 cells allergies are caused by characteristic immunoresponses to allergens, primarily mediated by Th2 cells. Th2 cells synthesize high levels of interleukin 4 (IL-4), IL-5, and IL-13, which leads to the release of allergen-specific immunoglobulin E (IgE) and the production of mediators from mast cells [3]. It has largely been aceepted that an imbalance between Th1 and Th2 cells leads to the clinical expression of this allergic disease [4]. Spina *et al*. reported that the pathophysiology of asthma is related to a Th1/Th2 cell imbalance in the airways [5]. Because asthma is associated with decreased Th2 responses, enhanced Th1 responses may suppress the development of allergic airway inflammation.

Lung inflammation in allergic asthma is induced by a cascade of reactions involving several mediators, including nitric oxide (NO). NO plays an essential role in regulating airway function and in the pathophysiology of inflammatory airway diseases. NO is synthesized from L-arginine by NO synthase (NOS), which exists in three isoforms: neuronal NOS (nNOS: NOS-1), endothelial NOS (eNOS: NOS-3), and inducible NOS (iNOS: NOS-2). It is now known that each of these isoforms may be expressed in a variety of tissues and cell types [6]. However, iNOS is induced after exposure to proinflammatory cytokines and is expressed in epithelial and inflammatory cells of the airway [7]. Several studies have suggested that the downregulation of iNOS expression inhibits airway inflammation in an asthmatic model [8,9].

Traditional herbal medicines are composed of various components. These components exhibit various therapeutic effects; thus, mixed herbal medicines are applied to multiple diseases [10]. Samsoeum (known as Shen-su-yin in Chinese) is composed of 12 different crude components obtained from natural herbs. It is one of the major traditional herbal medicines that are used widely in the treatment of headache, fever, cough and rhinorrhea caused by cold wind. According to previous studies, Samsoeum has immunomodulating [11] and antiallergic effects [12]. However, there is little information on the mechanism underlying the antiasthmatic effect of Samsoeum. In particular, the inhibitory mechanism of Samsoeum on airway inflammation remains unclear. Therefore, we examined the protective effects of a Samsoeum water extract (SSEW) on the airway inflammation induced by ovalbumin (OVA) challenge.

## Methods

### Preparation of the SSEW

A voucher specimen of Samsoeum (2008-KE28) is available at the Herbal Medicine Formulation Research Group, Korea Institute of Oriental Medicine. Samsoeum was prepared in our laboratory from a mixture of chopped crude herbs that were purchased from Omniherb (Korea; Geochang, Jecheon, Geumsan, Yeongcheon, and Jeju) and HMAX (China). Professor Je-Hyun Lee of Dongguk University, Gyeongju, Republic of Korea, confirmed the identity of each crude herb. A decoction of Samsoeum was prepared using a mixture of herbal medicines according to composition in the laboratory (Table 1). The herbal medicines of Samsoeum were extracted in distilled water at 100°C for 2 h. The extract solution was evaporated to dryness and then frozen to a dry powder (yield, 18.6%).

**Table 1:** **Crude components of Samsoeum**

**Scientific name**	**Amount (g)**	**Company of purchase**	**Source**
Perillae Herba	3.75 (9.1%)	Omniherb	Geochang, Korea
Puerariae Radix	3.75 (9.1%)	Omniherb	Jecheon, Korea
Pinelliae Tuber	3.75 (9.1%)	HMAX	China
Anthrisci Radix	3.75 (9.1%)	HMAX	China
Ginseng Radix	3.75 (9.1%)	Omniherb	Geumsan, Korea
Poria Sclerotium	3.75 (9.1%)	Omniherb	Yeongcheon, Korea
Aurantii Fructus	2.8125 (6.8%)	HMAX	China
Platycodonis Radix	2.8125 (6.8%)	Omniherb	Yeongcheon, Korea
Glycyrrhizae Radix	2.8125 (6.8%)	HMAX	China
Citri Unshius Pericarpium	2.8125 (6.8%)	Omniherb	Jeju, Korea
Zingiberis Rhizoma	3.75 (9.1%)	Omniherb	Yeongcheon, Korea
Zizyphi Fructus	3.75 (9.1%)	Omniherb	Yeongcheon, Korea
Total amount	41.25 (100%)		

### Animals

Specific pathogen-free female BALB/c mice (6 weeks of age) were purchased from Orient Co. (Seoul, Korea) and used after 1 week of quarantine and acclimatization. Mice were maintained in an animal facility under standard laboratory conditions for 1 week prior to experiments, and were provided with water and standard chow *ad libitum*. All experimental procedures were performed in compliance with the NIH guidelines for the Care and Use of Laboratory Animals and were approved by Korea Institute of Oriental Medicine Institutional Animal Care and Use Committee (Approval number: #10-011). The animal handling followed the dictates of the National Animal Welfare Law of Korea.

### OVA-induced allergic asthma

The mice were randomly divided into 5 groups (n = 6 per group): NC (normal control group, PBS sensitization/challenge), OVA (OVA sensitization/challenge), Mon (OVA sensitization/challenge + oral gavage of 30 mg/kg of montelukast), SSEW-100 (OVA sensitization/challenge + oral gavage of 100 mg/kg of SSEW), and SSEW-200 (OVA sensitization/challenge + oral gavage of 200 mg/kg of SSEW).

OVA sensitization and airway challenge were performed as described previously [13]. Briefly, mice were sensitized on days 0 and 14 by intraperitoneal injection of 20 μg of OVA emulsified in 2 mg of aluminum hydroxide in 200 μL of PBS buffer (pH 7.4). On days 21, 22, and 23 after the initial sensitization, mice received an airway challenge with OVA (1%, w/v, in PBS) for 1 h using an ultrasonic nebulizer (NE-U12; Omron Corp., Tokyo, Japan). SSEW was dissolved in PBS and was prepared fresh daily before each treatment. SSEW was administered by gavage to mice at doses of 100 mg/kg or 200 mg/kg once daily from days 18 to 23. Normal- and positive-control mice were administered PBS or Mon (30 mg/kg in PBS) orally, respectively. Mon was developed as a cysteinyl leukotriene (cys-LT)-1 receptor antagonist [14] and was introduced into the market after successful clinical evaluation in patients with aspirin-sensitive asthma, nocturnal exacerbation of asthma, and allergic asthma [15].

**Figure 1:**
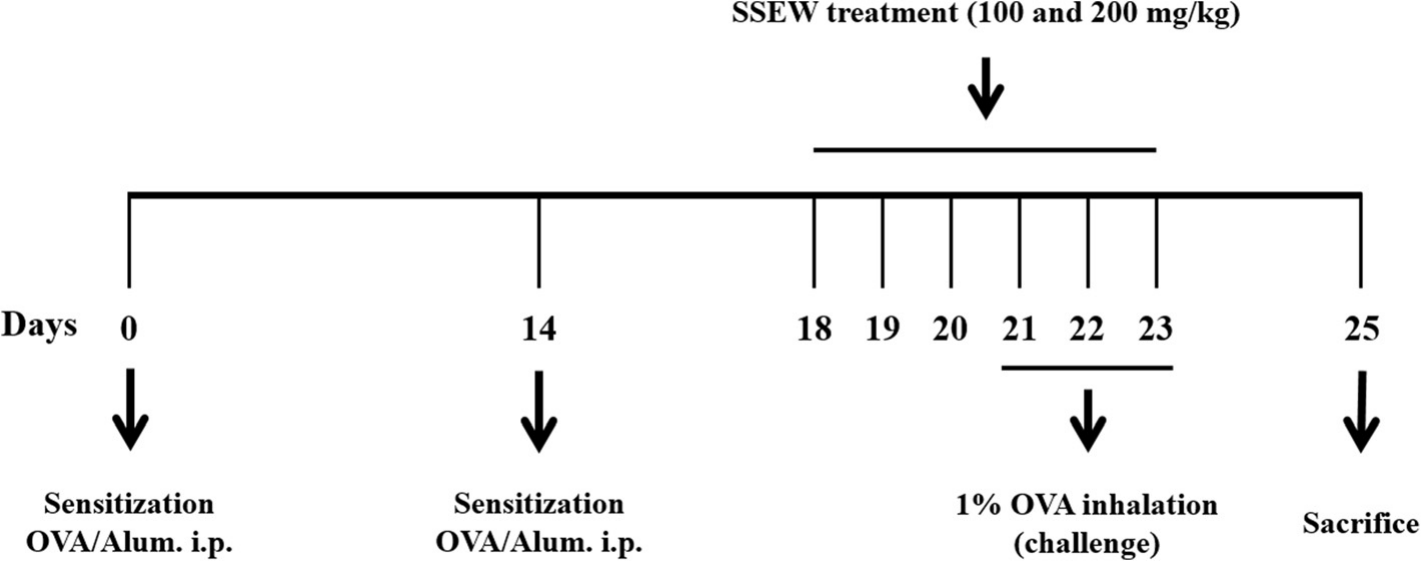
**Asthmatic mouse model: airway inflammation and treatment with SSEW.**

After the OVA challenge, bronchoalveolar lavage fluid (BALF) samples were obtained from the mice and processed, and inflammatory cells were counted as described previously [13]. Briefly, mice were sacrificed by intraperitoneal injection of pentobarbital (50 mg/kg; Hanlim Pharm. Co., Seoul, Korea) 48 h after the last challenge, and tracheostomy was performed. A schematic diagram of the treatment schedule is shown in Figure 1. To obtain BALF, ice-cold PBS (0.5 mL) was infused into the lung and withdrawn via tracheal cannulation three times (total volume, 1.5 mL). The number of total inflammatory cells was assessed by counting cells in at least five squares of a hemocytometer after exclusion of dead cells by Trypan blue staining. To determine differential cell counts, 100 μL of BALF was centrifuged onto slides using a cytospin machine (Hanil Science Industrial, Seoul, Korea) (200 g, 4°C, 10 min). The slides were dried and the cells were fixed and stained using the Diff-Quik® staining reagent (B4132-1A; IMEB Inc., Deerfield, IL), according to the manufacturer’s instructions. The supernatant obtained from BALF was stored at −70°C for biochemical analysis.

### Measurement of the levels of Th1/Th2 cytokines, chemokines, and IgE

The levels of TNF-α, eotaxin, IL-4, IL-5, IL-10, IL-13, and IL-33 in BALF were measured using enzyme-linked immunosorbent assay (ELISA) kits (BioSource International, Camarillo, CA) according to the manufacturer’s protocols. The detection range of IL-13 is 3.9 pg/mL to 250 pg/mL, and IL-5 is 7.8 pg/mL to 500 pg/mL. The detection ranges of TNF-α and IL-4 are 15.6 pg/mL to 1000 pg/mL, and eotaxin, IL-10 and IL-33 are 31.25 pg/mL to 2000 pg/mL. The levels of total IgE and OVA-Specific in BALF and plasma were measured using ELISA. Microtiter plates were coated with anti-IgE antibodies (anti-mouse IgE; 10 g/mL; Serotec, Oxford, UK) in PBS-Tween 20, and incubated with BALF or plasma. The plates were then washed four times, and 200 μL of O-phenylenediamine dihydrochloride (Sigma-Aldrich, St. Louis, MO) was added to each well. The plates were incubated for 10 min in the dark and the absorbance was then measured at 450 nm.

### Lung tissue histopathology

After BALF samples were obtained, lung tissue was fixed in 10% (v/v) neutral-buffered formalin. Tissues were embedded in paraffin, sectioned at a 4 μm thickness, and stained with hematoxylin and eosin stain (H&E) solution (hematoxylin, Sigma MHS-16, and eosin, Sigma HT110-1-32) and periodic acid–Schiff (PAS) (IMEB Inc., San Marcos, CA) to estimate inflammatory cell accumulation and mucus production, respectively. Quantitative analysis for airway inflammation and mucus secretion was evaluated using a MetaMorph Offline version 7.7.0.0 image analysis software (Molecular Devices Inc., Sunnyvale, CA, USA).

### Western blotting

Equal amounts of total lung protein (30 μg) were heated at 100°C for 5 min, loaded onto 8% sodium dodecylsulfate polyacrylamide gel electrophoresis gels, and electrophoresed. The proteins were then transferred to a nitrocellulose membrane (at 100 V for 2 h). The membrane was blocked for 1 h with Tris-buffered saline containing 0.05% Tween-20 (TBST) plus 5% skim milk, followed by incubation with anti-iNOS (sc-7271, 1:1000 dilution; Santa Cruz Biotechnology, Santa Cruz, CA) and anti-β-actin (#4967S, 1:1000 dilution; Cell Signaling Technology, Danvers, MA) antibodies overnight at 4°C. The embrane was washed three times with TBST at intervals of 10 min and then incubated with a horseradish peroxidase (HRP)-conjugated secondary antibody (iNOS: anti-mouse, β-actin: anti-rabbit 1:3000 dilution; Jackson ImmunoResearch, West Grove, PA) for 1 h at room temperature. The membrane was washed three times with TBST at intervals of 10 min and developed using an enhanced chemiluminescence kit (ECL; Amersham Pharmacia Biotech, Uppsala, Sweden). Then, membrane were photographed and for quantitative analysis, densitometric band values were determined using commercially available ChemiDoc™ XRS^+^ imaging system (Bio-Rad Laboratories, Hercules, CA, USA).

### Statistical analyses

Data are expressed as means ± standard error of the mean (SEM). Statistical significance was determined using analysis of variance (ANOVA) followed by a multiple comparison test with Bonferroni adjustment. *P* values < 0.05 or < 0.01 were considered significant.

## Results

### SSEW reduced the number of inflammatory cells in BALF

**Figure 2:**
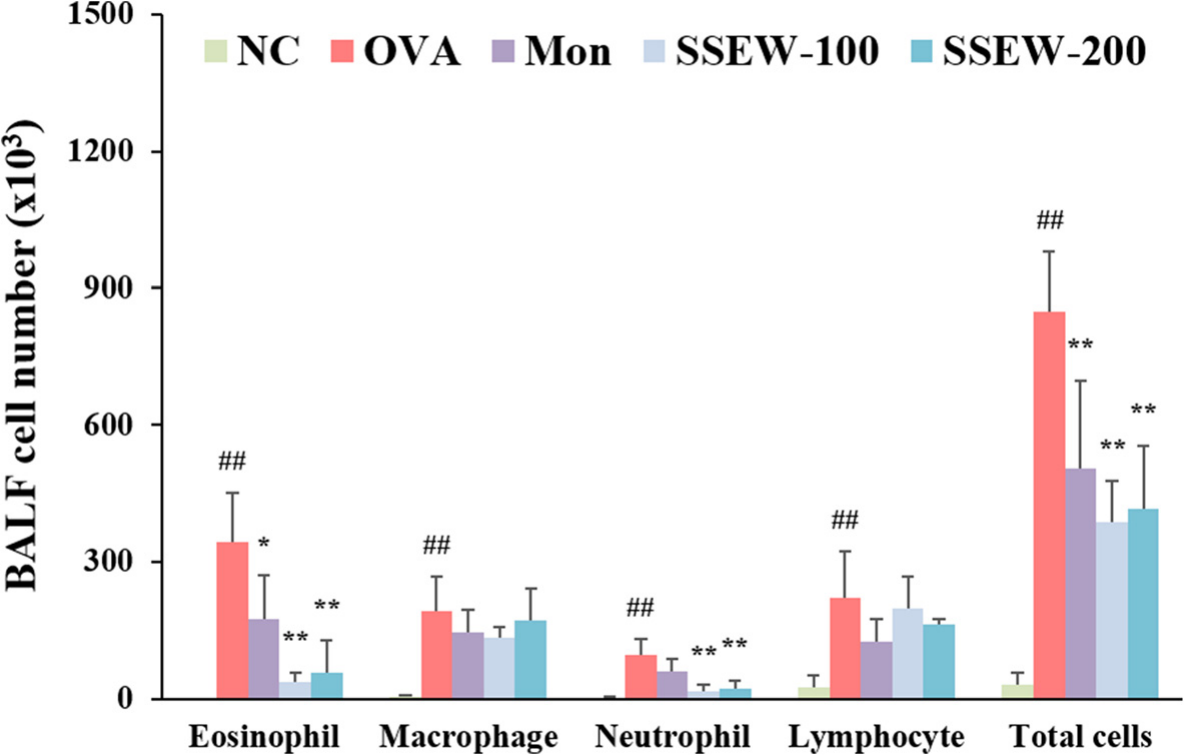
**Effect of SSEW on inflammatory cell accumulation in the BALF of OVA-induced mice.** BALF was collected 48 h after the last OVA challenge, and the cells were isolated by cytospin and stained with Diff-Quick. NC, normal control group (vehicle); OVA, OVA-induced group (control); Mon, montelukast (30 mg/kg) + OVA-induced group (positive control); SSEW-100, SSEW (100 mg/kg) + OVA-induced group; SSEW-200, SSEW (200 mg/kg) + OVA-induced group. The values represent the mean ± S.E.M (n = 6/group). Significant differences at ^##^ *P* < 0.01 compared with the NC group. Significant differences at *P < 0.05 and **P < 0.01 compared with the OVA-induced group.

To measure inflammatory cells, we counted the number of total cells, lymphocyte, neutrophils, macrophages, and eosinophils in BALF. The levels of inflammatory cells in BALF were significantly elevated in the OVA group compared with the NC group. However, SSEW treatment (100 and 200 mg/kg) markedly reduced the number of total cells, neutrophil and eosinophils than in the Mon group, which was a positive-control drug in this study (Figure 2).

### SSEW decreased the Th1 cytokine and Th2 chemokine levels in BALF

**Figure 3:**
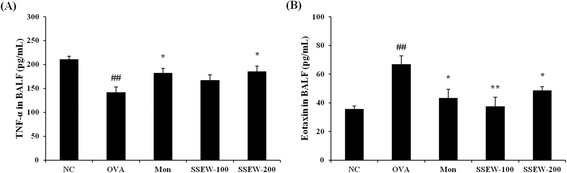
**Effect of SSEW on Th1 cytokines and Th2 chemokine the in BALF of OVA-induced mice.** Individual data were obtained using ELISA. **(A)** TNF-α and **(B)** eotaxin levels. NC, normal control group (vehicle); OVA, OVA-induced group (control); Mon, montelukast (30 mg/kg) + OVA-induced group (positive control); SSEW-100, SSEW (100 mg/kg) + OVA-induced group; SSEW-200, SSEW (200 mg/kg) + OVA-induced group. The values represent the mean ± S.E.M (n = 6/group). Significant differences at ^##^ *P* < 0.01 compared with the NC group. Significant differences at *P < 0.05 and **P < 0.01 compared with the OVA-induced group.

To investigate the effects of SSEW on the T-cell immune response in asthmatic mice, we evaluated the production of Th1-type cytokines and the Th2-type chemokines in BALF. As shown in Figure 3A, the level of TNF-α was significantly decreased in the OVA group compared with the NC group. However, the Mon group exhibited a significantly increased level of TNF-α compared with the OVA group, and the level of TNF-α increased gradually in the SSEW groups (100 and 200 mg/kg) compared with the OVA group in a dose-dependent manner. In contrast, the level of eotaxin was significantly increased in the OVA group compared with the NC group. Finally, the administration of Mon and SSEW (100 and 200 mg/kg) significantly inhibited the level of eotaxin compared with what was observed in the OVA group (Figure 3B).

### SSEW decreased the levels of Th2 cytokines in BALF

**Figure 4:**
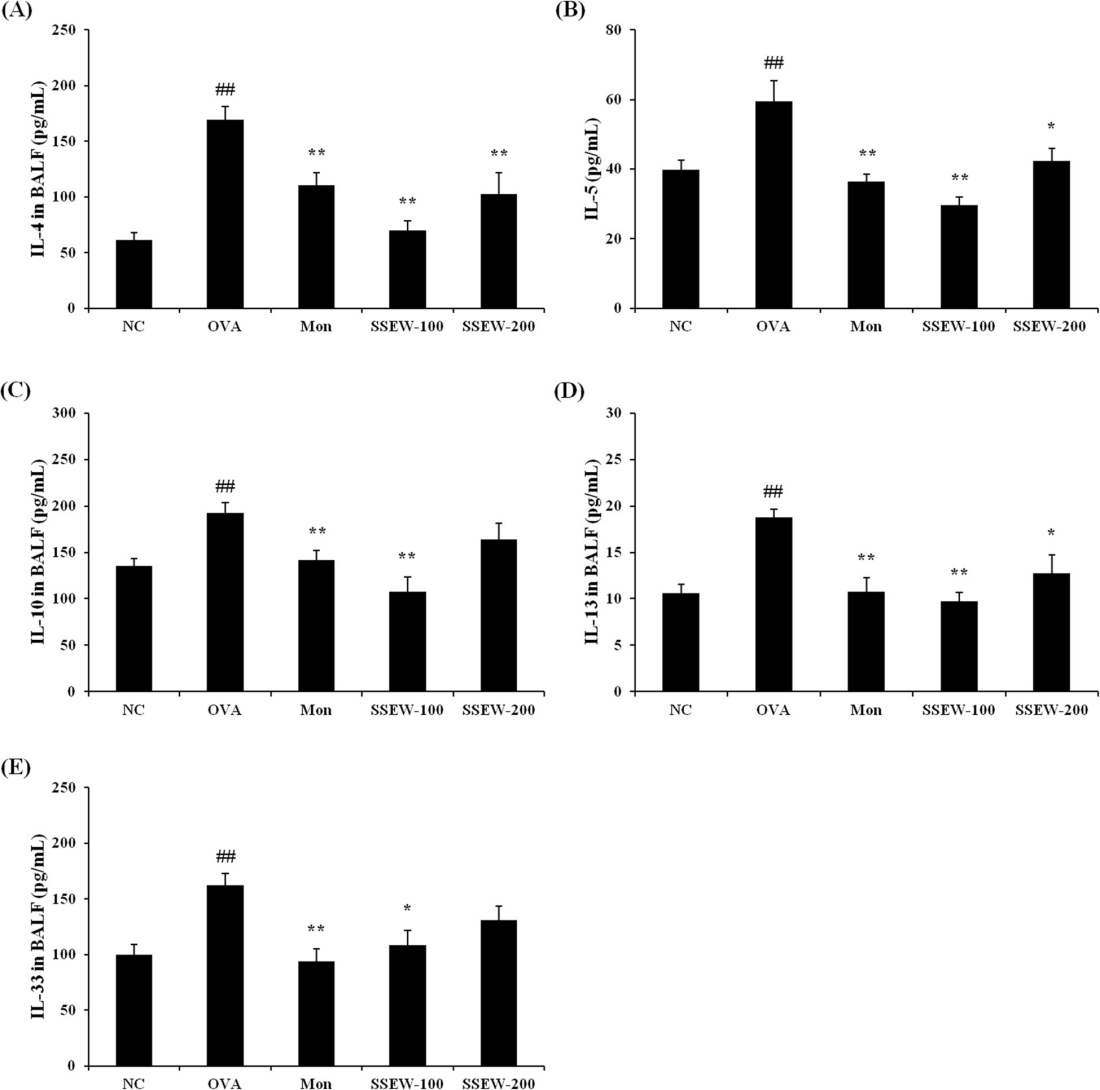
**Effect of SSEW on Th2 cytokines in the BALF of OVA-induced mice. Individual data were obtained using ELISA.** **(A)** IL-4, **(B)** IL-5, **(C)** IL-10, **(D)** IL-13, and **(E)** IL-33. NC, normal control group (vehicle); OVA, OVA-induced group (control); Mon, montelukast (30 mg/kg) + OVA-induced group (positive control); SSEW-100, SSEW (100 mg/kg) + OVA-induced group; SSEW-200, SSEW (200 mg/kg) + OVA-induced group. The values represent the mean ± S.E.M (n = 6/group). Significant differences at ^##^ *P* < 0.01 compared with the NC group. Significant differences at *P < 0.05 and **P < 0.01 compared with the OVA-induced group.

To confirm the association between SSEW and Th2 responses in BALF from OVA-induced asthmatic mice, we assessed the levels of Th2-type cytokines in BALF. Figure 4 shows that the OVA group exhibited significantly elevated levels of Th2-type cytokines, including (A) IL-4, (B) IL-5, (C) IL-10, (D) IL-13, and (E) IL-33, compared with the NC group. However, the SSEW groups (100 and 200 mg/kg) showed significantly decreased levels of Th2 cytokines compared with the OVA group, similar to the Mon group (Figure 4A–E).

### SSEW improved histopathological changes in lung tissue

**Figure 5:**
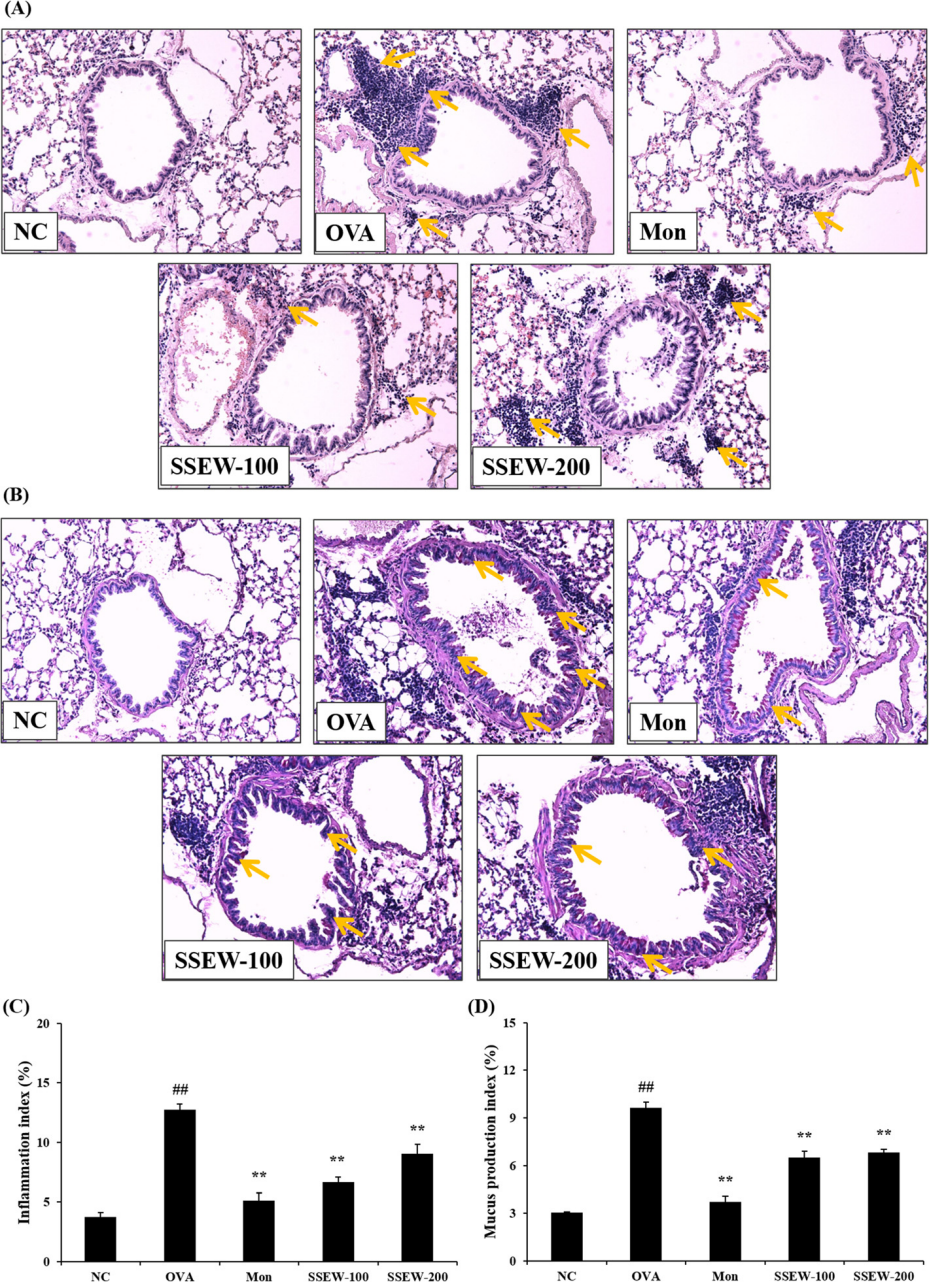
**Effect of SSEW on airway inflammation and airway goblet cell hyperplasia in the lung tissues of OVA-induced mice.** Lung tissues were stained with **(A)** H&E solution and **(B)** PAS. The panel is magnified (200 ×). Representative photomicrographs of lung sections are shown. Yellow arrows indicated as airway inflammation and mucus production. **(C)** Inflammation and **(D)** mucus production index were determined using an image analyzer, respectively. NC, normal control group (vehicle); OVA, OVA-induced group (control); Mon, montelukast (30 mg/kg) + OVA-induced group (positive control); SSEW-100, SSEW (100 mg/kg) + OVA-induced group; SSEW-200, SSEW (200 mg/kg) + OVA-induced group. The values represent the mean ± S.E.M (n = 3/group). Significant differences at ^##^ *P* < 0.01 compared with the NC group. Significant differences at **P < 0.01 compared with the OVA-induced group.

To analyze the effects of SSEW on the histological features of asthma, we performed H&E and PAS staining in lung tissues of OVA-induced asthmatic mice. OVA-induced lung tissues were characterized by peribronchial and perivascular inflammation caused by leukocyte infiltration compared with normal lung tissues; most infiltrating inflammatory cells were eosinophils. However, treatment with SSEW (100 and 200 mg/kg) markedly decreased eosinophil-rich leukocyte infiltration compared with the OVA group (Figure 5A,C). Mucus overproduction caused by goblet cell hyperplasia is characteristic of airway obstruction and airway remodeling. In the OVA group, mucus overproduction was clearly observed as a violet color in lung tissues compared with the NC group. However, administration of SSEW (100 and 200 mg/kg) markedly attenuated the mucus overproduction compared with the OVA group (Figure 5B,D).

### SSEW reduced the release of IgE levels in plasma and BALF

Because Th2-type cytokines promote airway inflammation in asthma via the increase in IgE levels, we evaluated the levels of IgE in plasma and BALF. As shown in Table 2, the levels of IgE were significantly increased in the OVA group (total IgE in BALF, 3.52 ± 0.36 ng/mL; total IgE and OVA-specific IgE in plasma, 1073.31 ± 123.77 and 54.57 ± 11.88; *P* < 0.01) compared with those observed in the NC group. However, SSEW treatment significantly inhibited the levels of total IgE (SSEW-100 and SSEW-200 in BALF, 2.73 ± 0.59 and 2.56 ± 0.37 ng/mL; SSEW-100 and SSEW-200 in plasma, 793.52 ± 83.49 and 771.48 ± 68.52 ng/mL) and OVA-specific IgE (SSEW-100 and SSEW-200 in plasma, 32.84 ± 15.23 and 45.35 ± 7.67 ng/mL) compared with the OVA group.

**Table 2:** **Effect of SSEW on IgE levels in BALF and plasma**

**Groups**	**BALF**	**Plasma**	
	**Total IgE (ng/mL)**	**Total IgE (ng/mL)**	**OVA-specific IgE (ng/mL)**
NC	1.95 ± 10.63	262.95 ± 107.60	< 0
OVA	3.52 ± 0.36^##^	1073.31 ± 123.77^##^	54.57 ± 11.88^##^
Mon	2.24 ± 0.50^**^	636.29 ± 102.28**	33.32 ± 10.35**
SSEW-100	2.73 ± 0.59^*^	793.52 ± 83.49**	32.84 ± 15.23*
SSEW-200	2.56 ± 0.37^**^	771.48 ± 68.52**	45.35 ± 7.67

NC, normal control group (vehicle); OVA, OVA-induced group (control); Mon, montelukast (30 mg/kg) + OVA-induced group (positive control); SSEW-100, SSEW (100 mg/kg) + OVA-induced group; SSEW-200, SSEW (200 mg/kg) + OVA-induced group. The values represent the mean ± S.E.M. (n = 6 in each group). Significant differences at ^##^ *P* < 0.01 compared with the NC group. Significant differences at *P < 0.05 and **P < 0.01 compared with the OVA-induced group.

### SSEW inhibited the expression of iNOS in lung tissue

**Figure 6:**
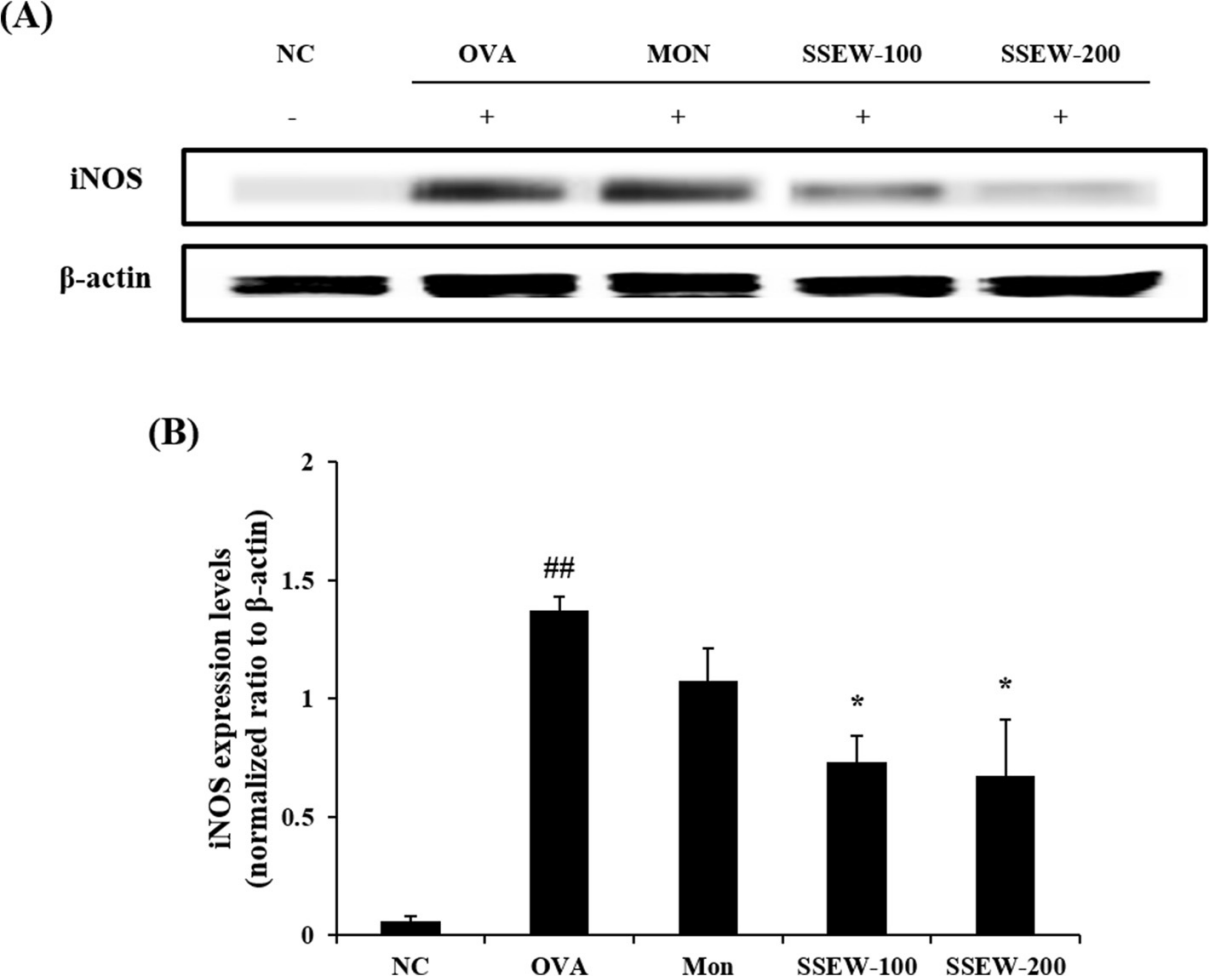
**Effect of SSEW on iNOS expression in lung tissues of OVA-induced mice.** Lung tissues were homogenized and iNOS protein expression was determined using western blot analysis. **(A)** Expression of iNOS in the lung tissue. **(B)** Relative ratio: The relative levels of iNOS expression (normalized to β-actin) were quantified. NC, normal control group (vehicle); OVA, OVA-induced group (control); Mon, montelukast (30 mg/kg) + OVA-induced group (positive control); SSEW-100, SSEW (100 mg/kg) + OVA-induced group; SSEW-200, SSEW (200 mg/kg) + OVA-induced group. The values represent the mean ± S.E.M (n = 3/group). Significant differences at ^##^ *P* < 0.01 compared with the NC group. Significant differences at *P < 0.05 compared with the OVA-induced group.

To assess the association between the expression of the iNOS protein and bronchial asthma, we evaluated iNOS expression by western blotting. iNOS was markedly upregulated in the OVA group compared with the NC group. SSEW treatment (100 and 200 mg/kg) suppressed the upregulation of iNOS in a dose-dependent manner (Figure 6).

## Discussion

In the present study, we investigated the anti-inflammatory and anti-asthmatic effects of SSEW in an OVA-induced murine asthma model. OVA-induced mice showed an increased number of inflammatory cells, elevated IgE production, imbalance of Th1/Th2 cytokines and chemokines in BALF, mucus hypersecretion, eosinophil-rich leukocyte infiltration, and increased expression of iNOS in lung tissues. However, SSEW treatment increased the level of a Th1 cytokine (TNF-α) and decreased the levels of Th2 cytokines (IL-4, IL-5, IL-10, IL-13, and IL-33) and of a chemokine (eotaxin), IgE production, and the number of inflammatory cells (particularly, eosinophils and neutrophil) in BALF. The administration of SSEW also inhibited mucus overproduction, leukocyte infiltration, and expression of iNOS in lung tissues. First, we demonstrated that SSEW decreased the expression of iNOS in this model.

In traditional herbal medicine, herb mixtures are considered to produce a synergistic effect and a reduction of side effects via the interaction between the various herbs [16]. SSEW is a water extract that consists of Perillae Herba, Puerariae Radix, Pinelliae Tuber, Anthrisci Radix, Ginseng Radix, Poria Sclerotium, Aurantii Fructus, Platycodonis Radix, Glycyrrhizae Radix, Citri Unshius Pericarpium, Zingiberis Rhizoma, and Zizyphi Fructus (Table 1). Among these herbs, earlier studies reported that Perillae Herba, Pinelliae Tuber and Platycodonis Radix have antiasthmatic effects [17-19]; Puerariae Radix, Poria Sclerotium, and Aurantii Fructus have the anti-inflammatory effects [20-22]; Glycyrrhizae Radix, Zingiberis Rhizoma, and Zizyphi Fructus have antioxidant effects [23-25]; Anthrisci Radix and Citri Unshius Pericarpium have antiobesity effects [26,27]; and Ginseng Radix has an immunomodulating effect [28]. Accordingly, we predicted that SSEW, which is a mixture of these herbs, would have pharmacological effects, including antiasthmatic and anti-inflammatory effects, on OVA-induced airway inflammation in a murine asthma model.

The infiltration of eosinophils into the airways is a characteristic of asthma, and increased numbers of these cells have been found in BALF. The proinflammatory mediators derived from eosinophils are major contributors to the inflammation observed in asthma, which includes airway epithelial cell loss and damage, airway dysfunction, and mucus hypersecretion [29]. Our results demonstrated that the administration of SSEW significantly reduced the number of eosinophil-rich leukocytes and neutrophil in BALF. Histopathological examination of lung tissues paralleled the results of the analysis of the cell count in BALF. In support of these results, histopathological findings revealed that SSEW treatment markedly attenuated the number of infiltrated inflammatory cells and mucus hyperproduction of goblet cells in lung tissues. Eosinophils regulate the allergen-dependent Th2 immune responses mediated by dendritic cells and T lymphocytes and attenuate Th1 responses [30]. Hence, the imbalance of Th1/Th2 cell responses plays an important part in the development of asthma, and induces different immune responses and immunologically dysregulated states. Th1 cells produce proinflammatory cytokines; among them, TNF-α is a potent multifunctional cytokine and a chemoattractant for neutrophils and eosinophils [31]. In our experiments, SSEW treatment significantly increased the reduced levels of TNF-α by OVA induction. Th2 cells play an important role in the initiation and progression of allergic diseases, including asthma. Th2 cytokines (IL-4, IL-5, IL-10, IL-13, and IL-33) and chemokine (eotaxin) induce airway inflammatory responses, such as infiltration of inflammatory cells, eosinophil activation, IgE production, and mucus hypersecretion [32]. Our findings indicate that SSEW administration significantly decreased the increased Th2-type cytokines and chemokine production. Several studies have shown that modulation of Th1/Th2 cytokines can inhibit airway inflammation in OVA-induced mice [33,34]. Therefore, these findings suggest that SSEW attenuates airway inflammation through the modulation of the Th1/Th2 balance.

IgE, which is a key target for the development of anti-asthma strategies, is one of the most important factors in the progression of allergic reactions. Previous studies have indicated that anti-IgE treatment may be effective in allergic diseases [13,35]. In our study, SSEW treatment significantly reduced the increase in the levels of IgE (total IgE and OVA-specific IgE) that was related to the development of allergic reactions. These results suggest that SSEW is an effective agent in the treatment of allergic asthma.

Excessive NO contributes to the pathogenesis of airway inflammation, cellular injury, and tissue damage in the lung. iNOS caused by NO production is well known for its potential role in inducing airway diseases, including asthma. iNOS production is increased in asthmatic lung tissues and several types of inflammatory cells [36]. Previous studies have shown that iNOS expression is closely related to the recruitment of inflammatory cells in airways, including eosinophils and neutrophils [9]. In the present study, the expression of the iNOS protein was intensely increased in lung tissues of OVA-induced mice. In contrast, SSEW markedly decreased the overexpression of iNOS induced by OVA in a dose-dependent manner. These findings indicate that SSEW may attenuate the infiltration of inflammatory cells via the downregulation of iNOS expression. Taken together, our results suggest that SSEW exerts potent antiasthmatic and anti-inflammatory effects by suppressing proinflammatory cytokines and mediators related to eosinophils.

## Conclusion

In summary, our results suggest that SSEW plays a pivotal role in OVA-induced airway inflammation by reducing the number of inflammatory cells (particularly, eosinophil and neutrophil), inflammatory cells infiltration, IgE levels, mucus production, and iNOS protein expression, and modulating Th1/Th2 cytokines. These findings suggest that SSEW has the potential to alleviate airway inflammation in the treatment of allergic asthma because of its antiasthma and anti-inflammatory properties.
